# Tetraphenylethene Derivatives Bearing Alkylammonium Substituents: Synthesis, Chemical Properties, and Application as BSA, Telomere DNA, and Hydroxyl Radical Sensors

**DOI:** 10.3390/molecules28155663

**Published:** 2023-07-26

**Authors:** Isao Yamaguchi, Kensuke Ikawa, Nobuto Takimiya, Aohan Wang

**Affiliations:** Department of Materials Chemistry, Shimane University, 1060 Nishikawatsu, Matsue 690-8504, Japann22m618@matsu.shimane-u.ac.jp (N.T.);

**Keywords:** tetraphenylethene, alkylammonium, sensor, TCNQ, BSA, DNA, hydroxyl radical

## Abstract

Tetraphenylethene derivatives (TPEs) are used as luminescence probes for the detection of metal ions and biomolecules. These sensors function by monitoring the increase in the photoluminescence (PL) intensity of the TPEs resulting from aggregation-induced emission (AIE) upon interaction with the analytes. The AIE behavior of the sensors was investigated by measuring their PL. In this study, PL, PL lifetime, and confocal laser scanning microscopy measurements were carried out as part of our in-depth investigation of AIE behavior of TPEs for the detection of biomolecules and radical species. We used 1,1,2,2-tetrakis(4-((trimethylammonium)alkoxy)phenyl)tetraphenylethene tetrabromide (TPE-C(*m*)N^+^Me_3_Br^−^, *m* = 2, 4, and 6, where *m* denotes the number of methylene groups in the alkyl chain) and TPE-C(*m*)N^+^Me_3_TCNQ^−•^ (TCNQ^−•^ is the 7,7′,8,8′-tetracyanoquinodimethane anion radical) as luminescent probes for the detection of bovine serum albumin (BSA), DNA, and the hydroxyl radical (^•^OH) generated from Fenton’s reagent. The sensing performance of TPE-C(*m*)N^+^Me_3_Br^−^ for BSA and DNA was found to depend on the length of the alkyl chains (*m*). UV-vis and PL measurements revealed that the responses of TPE-C(*m*)N^+^Me_3_Br^−^ and TPE-C(4)N^+^TCNQ^−•^ to Fenton’s reagent depended on the solvent. The electrochemical properties of the TPE derivatives prepared in this study were additionally investigated via cyclic voltammetry.

## 1. Introduction

The aggregation-induced emission (AIE) of tetraphenylethene derivatives (TPEs) is the result of the inhibition of bond rotation between the phenyl and ethene groups when these derivatives form aggregates [1,2,3]. TPE derivatives are used as luminescence probes for the detection of metal ions as well as biomolecules such as bovine serum albumin (BSA) and DNA [4,5,6,7,8]. These sensors operate by monitoring the AIE behavior attributable to the interaction between the TPE derivatives and analytes.

BSA has been extensively studied owing to its structural homology with human serum albumin. Tang reported that an anionic TPE derivative, 1,2-bis(4-(3-sulfonatoproxyl)phenyl)-1,2-diphenylethene sodium salt (**1**) [9], and a cationic TPE derivative, 1,2-bis[(4-((trimethylammonium)ethyl)phenyl)-1,2-diphenylethene dibromide (**2**) [10], could detect BSA by monitoring the increase in the photoluminescence (PL) intensity upon the addition of BSA to solutions of these TPE derivatives. Specifically, the anionic and cationic TPE derivatives interact electrostatically with the ammonium cation and carboxyl anion of BSA, respectively. The increase in the PL intensity of **1** and **2** upon the addition of BSA is the result of AIE. In this study, 1,1,2,2-tetrakis(4-((trimethylammonium)alkoxy)phenyl)tetraphenylethene tetrabromide (TPE-C(*m*)N^+^Me_3_Br^−^; *m* = 2, 4, and 6, where *m* denotes the number of methylene groups in the alkyl chain) was used for the detection of BSA. TPE-C(*m*)N^+^Me_3_Br^−^ is expected to interact electrostatically with BSA via its four ammonium groups, thereby inhibiting the rotation of all of the bonds between the ethene and phenyl groups. This inhibition is anticipated to give rise to effective AIE and enable analytes to be detected with high sensitivity. Because the PL lifetimes of π-conjugated molecules have been reported to increase when they aggregate in solution [11,12], PL lifetime (t’s) measurements and confocal laser scanning microscopy (CLSM) analyses of the TPE-C(*m*)N^+^Me_3_Br^−^ solutions were conducted before and after the addition of BSA. This information was expected to advance our understanding of the AIE behavior of the TPE derivatives when they are used as sensing probes.

In addition to the detection of BSA, we investigated the telomere DNA sensing behavior of TPE-C(*m*)N^+^Me_3_Br^−^. Telomere DNA is an essential part of human chromosomes that regulates cell ageing. Human telomere DNA is composed of a repeating sequence of nucleotides, namely TTAGGG (T = thymine, A = adenine, and G = guanine). Guo reported that 1,1,2,2-tetrakis(4-((trimethylammonium)butyl)phenyl)tetraphenylethene tetrabromide (TPE-C(4)N^+^Me_3_Br^−^) could detect the chain lengths of GGG(TTAGGG)*_n_* (*n* = 3, 7, 8, 9, 10, and 11). This sensor is based on the occurrence of AIE upon the addition of DNA to the TPE-C(4)N^+^Me_3_Br^−^ solution [13,14]. However, with the exception of TPE-C(4)N^+^Me_3_Br^−^, TPE-C(*m*)N^+^Me_3_Br^−^ has not been used for DNA sensing. The length of the alkyl chain (*m*) of TPE-C(*m*)N^+^Me_3_Br^−^ is likely to affect the interaction between the ammonium group of TPE-C(*m*)N^+^Me_3_Br^−^ and the phosphate group of DNA. With this in mind, we investigated the extent to which the length of the alkyl chain of TPE-C(*m*)N^+^Me_3_Br^−^ affects the PL intensity and lifetime by measuring these quantities before and after the addition of (TTAGGG)*_n_* (*n* = 2, 4, and 6). These data are useful for understanding the AIE behavior of TPE derivatives with respect to DNA sensing.

Alternative applications of TPE-C(*m*)N^+^Me_3_Br^−^ (*m* = 2, 4, and 6) were explored by studying the anion exchange reaction of TPE-C(*m*)N^+^Me_3_Br^−^ with the lithium salt of the 7,7′,8,8′-tetracyanoquinodimethane anion radical (TCNQ^−•^). To the best of our knowledge, the products of this reaction, TPE-C(*m*)N^+^Me_3_TCNQ^−•^, are the first examples of TPE derivatives bearing TCNQ^−•^. TPE-C(*m*)N^+^Me_3_TCNQ^−•^ are expected to exhibit reactivity toward radical species owing to the presence of TCNQ^−•^. We previously reported that the TCNQ^−•^ group in cationic polymers acted as a terminator for the radical polymerization of methyl methacrylate and styrene, affording graft polymers with polymethylmethacrylate and polystyrene chains bonded to TCNQ^−^ [15].

The detection of the hydroxyl radical (^•^OH) has attracted attention because it is known to inflict damage upon biomolecules such as amino acids, lipids, and nucleic acids [16]. Several methods to detect ^•^OH with the help of fluorescent probes, such as coumarin derivatives [17,18,19,20,21], fluorescein derivatives [22], and fluorescent aromatic compounds with the 2,2,6,6-tetramethylpiperidine-1-oxyl moiety, have been reported [23]. Depending on the solvent, radical species such as ^•^OH and ^•^CH_3_ can be generated with the aid of Fenton’s reagent [24]. The detection of biomolecules and ^•^OH should preferably be conducted in water. However, the probes that were initially reported were used in organic solvents and mixtures consisting of water and an organic solvent because they were insoluble in water. In contrast, TPE-C(*m*)N^+^Me_3_Br^−^ can be used as sensing probes in both water and organic solvents because of their high solubilities. In this study, we investigated the PL and UV-vis responses of TPE-C(*m*)N^+^Me_3_Br^−^ and TPE-C(4)N^+^Me_3_TCNQ^−•^ to Fenton’s reagent. To the best of our knowledge, the use of TPE derivatives for the detection of radical species has not yet been reported. This work is expected to unveil fundamental information on the development of new probes based on TPE derivatives for the detection of radical species.

Herein, we report the synthesis, and the optical, and electrochemical properties of TPE-C(*m*)Br, TPE-C(*m*)N^+^Me_3_Br^−^, and TPE-C(*m*)N^+^Me_3_TCNQ^−•^ and their sensing performance of BSA, DNA, and radical species.

## 2. Results and Discussion

### 2.1. Synthesis

**Scheme 1:**
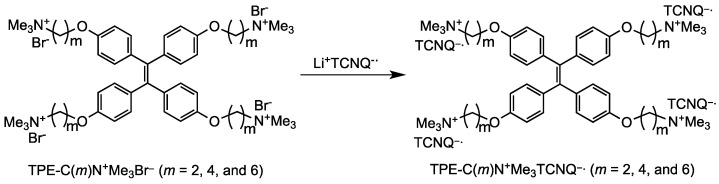
Synthesis of TPE-C(*m*)N^+^Me_3_TCNQ^−•^.

The reaction of TPE-C(*m*)N^+^Me_3_Br^−^ (*m* = 2, 4, and 6) with the lithium salt of the 7,7′,8,8′-tetracyanoquinodimethane anion radical (Li^+^TCNQ^−•^) proceeds via anion exchange between Br^−^ and TCNQ^−•^ to yield TPE-C(*m*)N^+^Me_3_TCNQ^−•^ (Scheme 1). Elemental analysis indicated that this anion exchange reaction proceeded to completion. TPE-C(*m*)N^+^Me_3_Br^−^ was soluble in water and polar organic solvents such as acetone, acetonitrile, and dimethyl sulfoxide (DMSO) but insoluble in tetrahydrofuran (THF), whereas TPE-C(*m*)N^+^Me_3_TCNQ^−•^ was soluble in the polar organic solvents but insoluble in water and THF. The IR absorption band corresponding to n(CN) of TPE-C(4)N^+^Me_3_TCNQ^−•^ was observed at 2178 cm^−1^ (Figure S1). This wavenumber is comparable to that of Et_4_N^+^TCNQ^−•^ (n = 2182 cm^−1^ [25]). The chemical shifts of the ^1^H NMR peaks corresponding to the TPE and alkyl protons of TPE-C(*m*)N^+^Me_3_TCNQ^−•^ were almost the same as those of TPE-C(*m*)N^+^Me_3_Br^−^. However, the ^1^H NMR signal corresponding to the TCNQ^−•^ protons of TPE-C(*m*)N^+^Me_3_TCNQ^−•^ disappeared due to the presence of the anion radical (Figure S2).

### 2.2. Optical Properties

**Figure 1:**
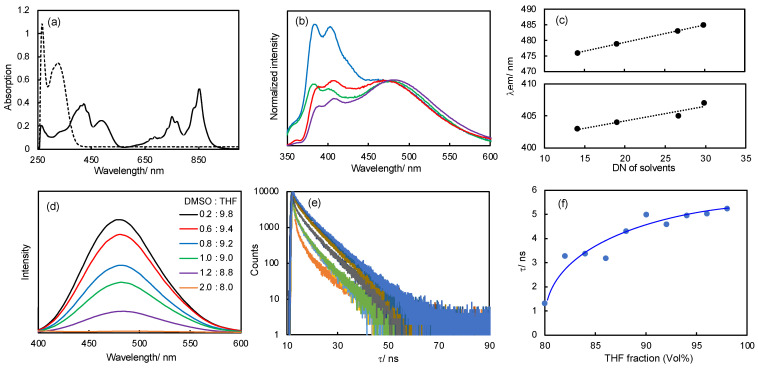
(**a**) UV-vis spectra of TPE-C(4)N^+^Me_3_Br^−^ (hashed curve) and TPE-C(4)N^+^Me_3_TCNQ^−•^ (solid curve) in DMSO. (**b**) PL spectra of TPE-C(4)N^+^Me_3_Br^−^ in acetonitrile (blue curve, DN = 14.1), methanol (green curve, DN = 19), *N*,*N*-dimethylformamide (red curve, DN = 26.6), and DMSO (purple curve, DN = 29.8). (**c**) Plots for λem vs. DN of solvents. (**d**) PL spectra of TPE-C(4)N^+^Me_3_Br^-^ in DMSO/THF. (**e**) Emission decay profiles of TPE-C(4)N^+^Me_3_Br^−^ in DMSO/THF. (**f**) THF fraction dependence of TPE-C(4)N^+^Me_3_Br^−^ emission lifetime in DM SO/THF. [TPE-C(4)N^+^Me_3_Br^−^] = 5.0 × 10^−5^ M.

Figure 1a shows the UV-vis spectra of TPE-C(4)N^+^Me_3_Br^−^ and TPE-C(4)N^+^Me_3_TCNQ^−•^ in DMSO. The UV-vis spectrum of TPE-C(4)Br closely resembles that of TPE-C(4)N^+^Me_3_Br^−^. The absorptions corresponding to the π−π* transitions of the phenyl rings and the TPE core of TPE-C(4)N^+^Me_3_Br^−^ were observed near 270 and 320 nm, respectively. In addition to these absorptions, TPE-C(4)N^+^Me_3_TCNQ^−•^ exhibited absorptions corresponding to the D_0_ → D_2_ and D_0_ → D_1_ transitions of the TCNQ^−•^ group in the ranges of 400−480 nm and 680−850 nm, respectively. These absorption ranges are similar to those of Et_3_NH^+^TCNQ^−•^ [26]. It appears that the alkyl chain of TPE-C(*m*)N^+^Me_3_Br^−^ and TPE-C(*m*)N^+^Me_3_TCNQ^−•^ hardly affects their optical properties as the UV-vis spectra of TPE-C(*m*)N^+^Me_3_Br^−^ and TPE-C(*m*)N^+^Me_3_TCNQ^−•^ are similar irrespective of the alkyl chain length (*m*), as shown in Figure S3. Figure 1b shows the photoluminescence (PL) spectra of TPE-C(4)N^+^Me_3_Br^−^ in various organic solvents. The PL peak positions shifted to longer wavelengths as the donor number (DN) of the solvents increased (Figure 1c). The results indicate that the geometry of the excited state could be different from that of the ground state. The bathochromic shift of the PL peak of TPE-C(4)-N^+^Me_3_Br^−^ appeared to be due to its increased stability in the excited state relative to that in the ground state. Furthermore, a large amount of stabilization energy produced via the solvation of TPE-C(4)-N^+^Me_3_Br^−^ may contribute to the bathochromic shift as the DN of the solvents increases. Figure 1d shows the PL spectra of TPE-C(4)N^+^Me_3_Br^−^ in DMSO and DMSO/THF mixtures. The PL intensity of TPE-C(4)N^+^Me_3_Br^−^ increases as the THF content of DMSO/THF increases. The increase in the PL intensity corresponds to the AIE, attributed to the immiscibility of TPE-C(4)N^+^Me_3_Br^−^ with THF. The confirmation that TPE-C(4)N^+^Me_3_Br^−^ underwent AIE was evident from the longer PL lifetimes (t’s) of TPE-C(4)N^+^Me_3_Br^−^ with an increase in the THF content of DMSO/THF (Figure 1f). The fluorescence decay profiles are shown in Figure 1e. As mentioned above, the PL lifetime (t) of π-conjugated molecules increases with aggregation [11,12]. Contrary to TPE-C(4)N^+^Me_3_Br^−^, TPE-C(4)N^+^Me_3_TCNQ^−•^ dissolved in DMSO did not undergo AIE in DMSO/THF. This result corresponds to the assumption that bulky TCNQ^−•^ groups prevent the aggregation of TPE-C(4)N^+^Me_3_TCNQ^−•^ in solutions.

### 2.3. Electrochemical Properties

**Figure 2:**
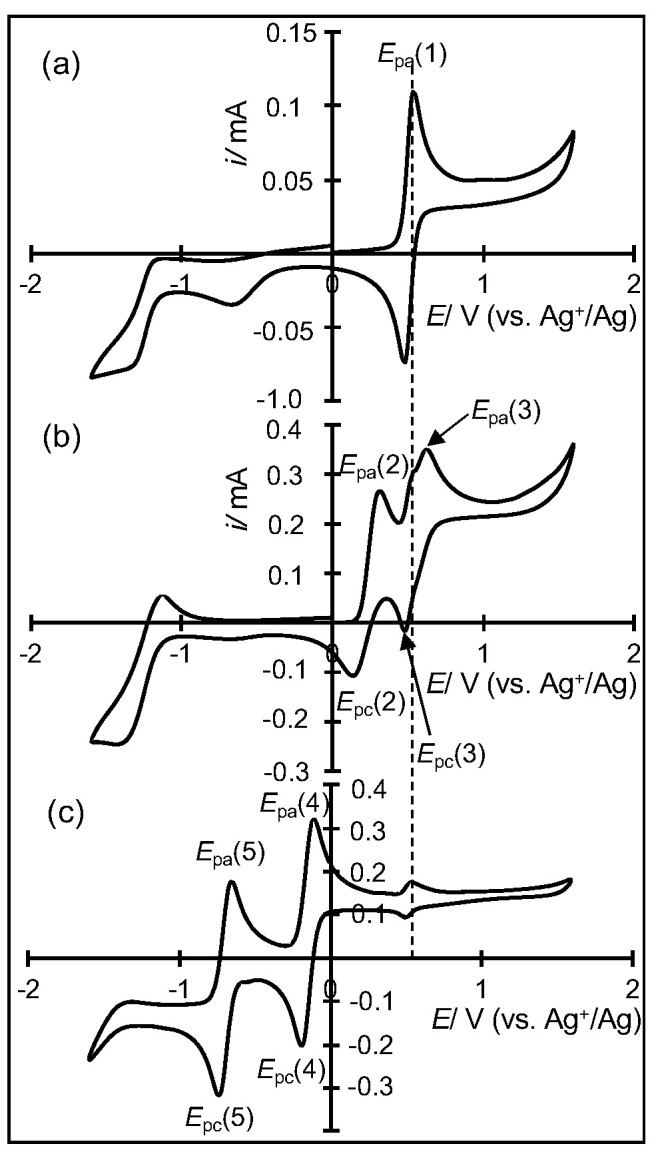
CV curves of (**a**) TPE-C(4)Br, (**b**) TPE-C(4)N^+^Me_3_Br^−^, and (**c**) TPE-C(4)N^+^Me_3_TCNQ^−•^ in acetonitrile containing 0.10 M [Et_4_N]BF_4_.

**Scheme 2:**
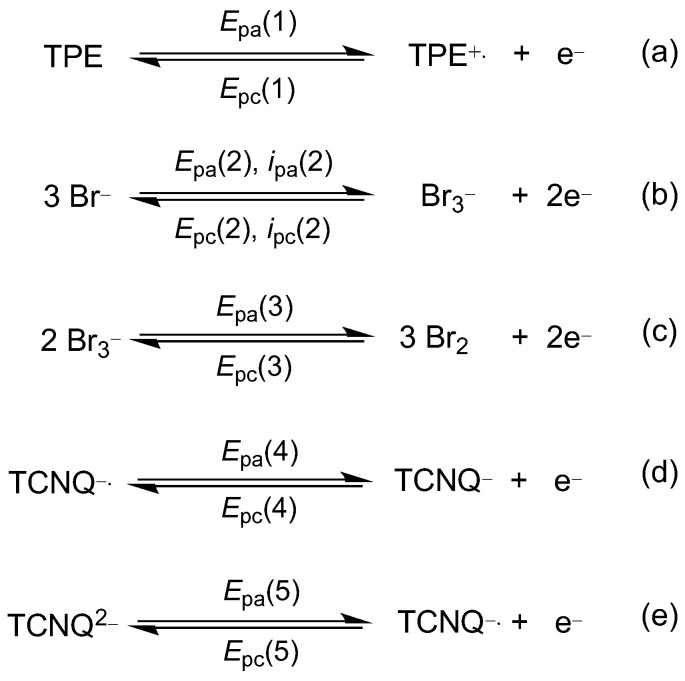
Electrochemical oxidation and reduction reactions in TPE-C(*m*)Br, TPE-C(*m*)N^+^Me_3_Br^−^, and TPE-C(*m*)N^+^Me_3_TCNQ^−•^.

The electrochemical properties of TPE-C(*m*)Br, TPE-C(*m*)N^+^Me_3_Br^−^, and TPE-C(*m*)N^+^Me_3_TCNQ^−•^ were investigated via cyclic voltammetry (CV). Figure 2a shows the CV curves of TPE-C(4)Br, TPE-C(4)N^+^Me_3_Br^−^, and TPE-C(4)N^+^Me_3_TCNQ^−•^ in acetonitrile containing 0.10 M [Et_4_N]BF_4_. The TPE core of TPE-C(4)Br underwent electrochemical oxidation at *E*_pa_(1) = 0.54 V (vs. Ag^+^/Ag). The CV curve of TPE-C(4)N^+^Me_3_Br^−^ shows two pairs of peaks, which correspond to the redox reaction between Br^−^ and Br_3_^−^ at *E*_pa_(2) = 0.31 V (vs. Ag^+^/Ag), and between Br_3_^−^ and Br_2_ at *E*_pa_(3) = 0.62 V (vs. Ag^+^/Ag). In addition, a shoulder peak corresponding to the electrochemical oxidation of the TPE core at *E*_pa_(1) = 0.55 V (vs. Ag^+^/Ag) is also observed. The *E*_pa_(2) and *E*_pa_(3) values of TPE-C(*m*)N^+^Me_3_Br^−^ (*m* = 2 and 6) were 0.31 V and 0.66 V (vs. Ag^+^/Ag) and 0.31 V and 0.62 V (vs. Ag^+^/Ag), respectively. The electrochemical reactions of the TPE derivatives are shown in Scheme 2. Reportedly, the two-step electrochemical oxidation of Br^−^ of tetraethylammonium bromide yields Br_2_ in solution [27]. Figure S3 shows plots of the peak currents of the oxidation of Br^−^ to Br_3_^−^ (*i*_pa_(2)) and reduction of Br_3_^−^ to Br^−^ (*i*_pc_(2)) vs. *v*^1/2^. Plotting the *i*_pa_(2) and *i*_pc_(2) values as a function of *v*^1/2^ revealed a linear relationship, indicating that the redox reaction is diffusion-controlled [28]. The TCNQ^−•^ group of TPE-C(4)N^+^Me_3_TCNQ**^−^^•^** undergoes a two-step electrochemical redox reaction (Scheme 2d,e). The CV curves of TPEC(*m*)N^+^Me_3_TCNQ**^−^^•^** (*m* = 2 and 6) are shown in Figure S5. The electrochemical reduction potentials of the TCNQ^−•^ group in TPE-C(4)N^+^Me_3_TCNQ**^−^^•^** are *E_pc_*(4) = –0.19 V and *E_pc_*(5) = –0.74 V (vs. Ag^+^/Ag). The *E_pc_*(4) and *E_pc_*(5) values of TPEC(*m*)N^+^Me_3_TCNQ**^−^^•^** (*m* = 2 and 6) were *E_pc_*(4) = –0.20 V and *E_pc_*(5) = –0.74 V (vs. Ag^+^/Ag) and *E_pc_*(4) = –0.18 V and *E_pc_*(5) = –0.75 V (vs. Ag^+^/Ag), respectively. These values are lower than the corresponding *E_pc_* values of TCNQ (*E_pc_* = –0.094 and –0.64 V (vs. Ag^+^/Ag) [29]) in a solution of tetrabutylammonium perchlorate in acetonitrile. This result corresponds to the fact that the TCNQ^−•^ moiety in TPE-C(*m*)N^+^Me_3_TCNQ**^−^^•^** is electrostatically bonded to the ammonium group.

### 2.4. BSA Sensing

**Figure 3:**
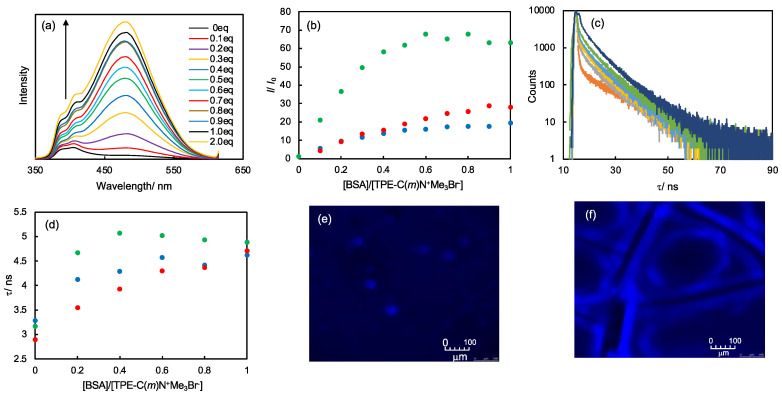
(**a**) PL spectral changes of TPE-C(4)N^+^Me_3_Br^−^ upon the addition of BSA in every 0.1 equimolar amounts in water. (**b**) BSA concentration dependence of *I*/*I*_0_ of TPE-C(*m*)N^+^Me_3_Br^−^ (*m* = 2: blue plots, 4: red plots, and 6: green plots). (**c**) Emission decay profiles of TPE-C(4)N^+^Me_3_Br^−^ in the presence of BSA in the absence (orange curve) and presence of BSA in 0.2 (gray curve), 0.4 (yellow curve), 0.6 (blue curve), 0.8 (green curve), and 1.0 (deep blue curve) equimolar amounts of BSA. (**d**) BSA concentration dependence of emission lifetime of TPE-C(*m*)N^+^Me_3_Br^−^ (*m* = 2: blue plots, 4: red plots, and 6: green plots). CLSM images of (**e**) TPE-C(4)N^+^Me_3_Br^−^ powder and (**f**) TPE-C(4)N^+^Me_3_Br^−^ in a cast film of BSA on glass plates. [TPE-C(4)N^+^Me_3_Br^−^] = 2.0 × 10^−5^ M.

Figure 3a depicts the changes in the PL spectra observed for TPE-C(4)N^+^Me_3_Br^−^ upon the addition of BSA in 0.1 equimolar amounts. The PL intensity of TPE-C(4)N^+^Me_3_Br^−^ increased with increasing amounts of BSA. The increase in the PL intensity upon the addition of BSA was also observed for TPE-C(*m*)N^+^Me_3_Br^−^ (*m* = 2 and 6) (Figure S4). Figure 3b shows the BSA concentration vs. the *I*/*I*_0_ values of TPE-C(*m*)N^+^Me_3_Br^−^, where *I*_0_ and *I* denote the PL intensity of TPE-C(*m*)N^+^Me_3_Br^−^ in the absence and presence of BSA, respectively.

These results show that TPE-C(*m*)N^+^Me_3_Br^−^ is able to successfully detect BSA in the concentration range of 0–4 × 10^−5^ M. This detection range is wider than that of **2** (0–8 × 10^−6^ M) [10]. The extent to which the PL increased in response to the addition of BSA increased with increasing alkyl chain length (*m*) of TPE-C(*m*)N^+^Me_3_Br^−^, as shown in Figure 3b. This result corresponds to the assumption that a long alkyl chain can prevent steric repulsion between the TPE core and BSA when the cationic group of TPE-C(*m*)N^+^Me_3_Br^−^ interacts with the anionic group of BSA. The images of the interactions between TPE-C(*m*)N^+^Me_3_Br^−^ (*m* = 2 and 6) and BSA or (GGGATT)*_n_* are shown in Figure S7. The PL lifetime and confocal laser scanning microscopy (CLSM) measurements revealed that the increase in the PL intensity of TPE-C(*m*)N^+^Me_3_Br^−^ upon the addition of BSA was attributed to AIE. The fluorescence decay profiles are shown in Figure 3c. Figure 3d shows the changes in the t values of an aqueous solution of TPE-C(*m*)N^+^Me_3_Br^−^ upon the addition of BSA. The t values of the aqueous solution of TPE-C(*m*)N^+^Me_3_Br^−^ increased as the BSA content of the solution increased. This result indicates that TPE-C(*m*)N^+^Me_3_Br^−^ underwent aggregation when interacting with BSA. The samples for the CLSM measurements were prepared by casting aqueous solutions of TPE-C(4)N^+^Me_3_Br^−^ on the glass plates in the absence and presence of BSA. Figure 3e,f depicts CLSM images of the TPE-C(4)N^+^Me_3_Br^−^ powder (sample A) and TPE-C(4)N^+^Me_3_Br^−^ cast in a BSA film (sample B) on glass plates. The CLSM image of sample A shows low-intensity bright spots, whereas that of sample B exhibits intense bright bands, indicating that the AIE of TPE-C(4)N^+^Me_3_Br^−^ was the result of the interaction with BSA.

The conformations of BSA in aqueous solutions of TPE-C(*m*)N^+^Me_3_Br^−^ were investigated by measuring the viscosity. The conformation of a macromolecule in solution is related to the a exponent of the Mark–Houwink–Sakurada equation, [*η*] = *KM*^a^ and the values of polymers in the expanded state are larger than those in contracted or intertwined states. Moreover, the viscosity of BSA reportedly depends on its conformation in aqueous solutions [30]. The specific viscosities (*η*_sp_′s) of aqueous solutions of BSA (*c* = 2.0 g·dL^−1^) at pH 2.7, 7.4, and 10.0 are 0.16, 0.056, and 0.058 dL·g^−1^, respectively [30]. These data correspond to the fact that BSA has an expanded conformation in acidic solutions and a contracted conformation in neutral and basic solutions. The *η*_sp_ values of aqueous solutions of BSA (*c* = 2.0 g·dL^−1^) in the presence of equimolar amounts of TPE-C(*m*)N^+^Me_3_Br^−^ (*m* = 2, 4, and 6) were 0.083, 0.082, and 0.088 dL·g^−1^, respectively. These values are smaller than the *η*_sp_ value of the aqueous solution of BSA in the absence of TPE-C(*m*)N^+^Me_3_Br^−^ (*h*_sp_ = 0.10 dL·g^−1^). This result indicates that the conformation of BSA forms is slightly contracted when it interacts with TPE-C(*m*)N^+^Me_3_Br^−^.

### 2.5. DNA Sensing

**Figure 4:**
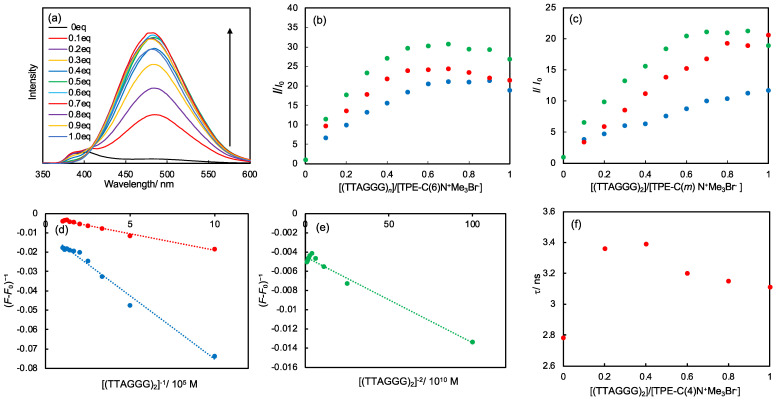
(**a**) PL spectral changes of TPE-C(4)N^+^Me_3_Br^−^ upon the addition of (TTAGGG)_6_ in 0.1 equimolar amounts. (**b**) (TTAGGG)*_n_* (*n* = 2 (blue plots), 4 (red plots), and 6 (green plots)) concentration dependence of *I*/*I*_0_ of TPE-C(6)N^+^Me_3_Br^−^. (**c**) Plots for *I*/*I*_0_ vs. [(TTAGGG)_2_]/[C(*m*)N^+^Me_3_Br^−^] (*m* = 2: blue plots, 4: red plots, and 6: green plots). (**d**) 1:1 Benesi–Hildebrand plots for TPE-C(2)N^+^Me_3_Br^−^ (blue plots) and TPE-C(4)N^+^Me_3_Br^−^ (red plots). (**e**) 1:2 Benesi–Hildebrand plots for TPE-C(6)N^+^Me_3_Br^−^. (**f**) (TTAGGG)_2_ concentration dependence of emission lifetime of TPE-C(4)N^+^Me_3_Br^−^. [TPE-C(*m*)N^+^Me_3_Br^−^] = 1.0 × 10^−5^ M.

**Figure 5:**
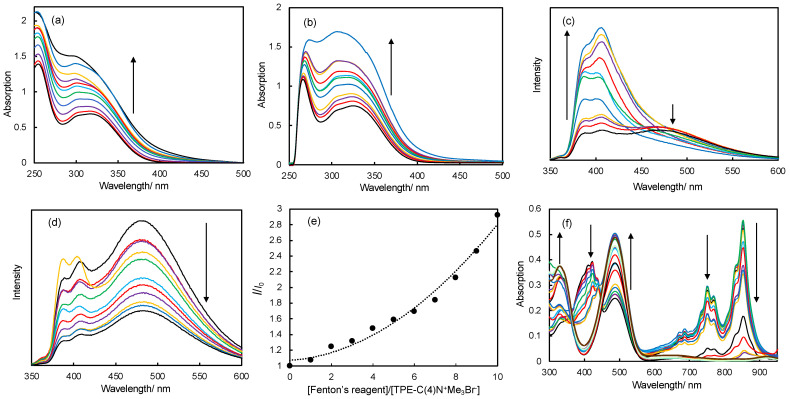
(**a**) UV-vis spectral changes of TPE-C(4)N^+^Me_3_Br^−^ with the addition of Fenton’s reagent in water in 1 equimolar amounts. (**b**) UV-vis spectral changes of TPE-C(4)N^+^Me_3_Br^−^ with the addition of Fenton’s reagent in DMSO. (**c**) PL spectral changes of TPE-C(4)N^+^Me_3_Br^−^ with the addition of Fenton’s reagent in water. (**d**) PL spectral changes of TPE-C(4)N^+^Me_3_Br^−^ with the addition of Fenton’s reagent in DMSO. (**e**) Stern–Volmer plots for TPE-C(4)N^+^Me_3_Br^−^ PL quenching using Fenton’s reagent. (**f**) UV-spectral changes of TPE-C(4)N^+^Me_3_TCNQ^−•^ with the addition of Fenton’s reagent in DMSO. [TPE-C(4)N^+^Me_3_Br^−^] = 5.0 × 10^−5^ M.

Figure 4a depicts the changes in the PL spectra of TPE-C(4)N^+^Me_3_Br^−^ upon the addition of (TTAGGG)_6_ in 0.1 equimolar amounts. The PL intensity of TPE-C(4)N^+^Me_3_Br^−^ increased until a 0.6 equimolar amount of DNA had been added and remained unchanged with the further addition of DNA. The PL intensity of TPE-C(*m*)N^+^Me_3_Br^−^ (*m* = 2 and 4) increased upon the addition of (TTAGGG)_6_ (Figure S5). These results indicate that TPE-C(*m*)N^+^Me_3_Br^−^ can detect DNA chain lengths in the six-base order, within several minutes, confirming that TPE-C(*m*)N^+^Me_3_Br^−^ significantly outperforms previously reported sensors. The DNA concentration vs. *I*/*I*_0_ of TPE-C(*m*)N^+^Me_3_Br^−^ is plotted in Figure 4b. The *I*/*I*_0_ values of TPE-C(*m*)N^+^Me_3_Br^−^ increased with increasing alkyl chain length (*m*). Figure 4c shows the (TTAGGG)_6_ concentration vs. *I*/*I*_0_ of TPE-C(*m*)N^+^Me_3_Br^−^. The *I*/*I*_0_ values of TPE-C(*m*)N^+^Me_3_Br^-^ upon the addition of DNA increased with increasing alkyl chain length (*m*). The results are comparable to the PL spectral changes of TPE-C(*m*)N^+^Me_3_Br^−^ with the addition of BSA. The *I*/*I*_0_ values of TPE-C(*m*)N^+^Me_3_Br^−^ (*m* = 2 and 4) increase in proportion to the amounts of DNA, whereas the *I*/*I*_0_ values of TPE-C(6)N^+^Me_3_Br^−^ increase until 0.5 equimolar amounts of DNA have been added, after which the values remain constant (Figure 5b). These results correspond to the assumption that TPE-C(*m*)N^+^Me_3_Br^−^ (*m* = 2 and 4) and TPE-C(6)N^+^Me_3_Br^−^ form complexes with DNA in 1:1 and 1:2 molar ratios, respectively. This assumption was confirmed via a Benesi–Hildebrand analysis using the following equations [31]:

1/(*F*_0_ − *F*) = 1/(*F*_0_ − *F*_∞_)*K*[DNA] + 1/(*F*_0_ − *F*_∞_)

1/(*F*_0_ − *F*) = 1/(*F*_0_ − *F*_∞_)*K*[DNA]^2^ + 1/(*F*_0_ − *F*_∞_)

where *F*_0_ and *F* denote the PL intensities of TPE-C(*m*)N^+^Me_3_Br^−^ in the absence and presence of DNA, respectively, and *F*_∞_ is the PL intensity at saturation. As shown in Figure 4d,e, straight lines were obtained by plotting the 1/(*F*_0_ − *F*) values as a function of 1/[(TTAGGG)_2_] in 1:1 Benesi–Hildebrand plots for TPE-C(*m*)N^+^Me_3_Br^−^ (*m* = 2 and 4), and by plotting the 1/(*F*_0_ − *F*) values as a function of 1/[(TTAGGG)_2_]^2^ in 1:2 Benesi–Hildebrand plots for TPE-C(6)N^+^Me_3_Br^−^. In contrast, plots of 1/(*F*_0_ − *F*) vs. 1/[(TTAGGG)_2_]^2^ in 1:2 Benesi–Hildebrand plots for TPE-C(*m*)N^+^Me_3_Br^−^ (*m* = 2 and 4), and of 1/(*F*_0_ − *F*) vs. 1/[(TTAGGG)_2_] in 1:1 Benesi–Hildebrand plots for TPE-C(6)N^+^Me_3_Br^−^ revealed that the relationship was nonlinear. Figure 4f shows the changes in the t values of aqueous solutions of TPE-C(*m*)N^+^Me_3_Br^−^ upon the addition of (TTAGGG)*_n_* (*n* = 2, 4, and 6). The PL lifetimes of TPE-C(*m*)N^+^Me_3_Br^−^ were lengthened after the addition of DNA. These results confirm that the increase in the PL of TPE-C(*m*)N^+^Me_3_Br^−^ upon the addition of DNA corresponds to AIE.

### 2.6. Hydroxyl Radical Sensing

Figure 5a,b shows the changes in the UV-vis spectra of TPE-C(4)N^+^Me_3_Br^−^ with the addition of Fenton’s reagent in water and DMSO, respectively. The absorption at approximately 270 nm corresponding to π–π* transitions of the phenyl rings of TPE-C(4)N^+^Me_3_Br^−^ increased with increasing amounts of Fenton’s reagent. However, the absorption at approximately 320 nm gradually decreased until four equimolar amounts of Fenton’s reagent had been added and disappeared with the addition of further amounts of Fenton’s reagent.

This result corresponds to the assumption that the reaction of radical species generated from Fenton’s reagent with the ethene group of TPE-C(4)N^+^Me_3_Br^−^ shortens the length of the π-conjugation. In this regard, it has been reported that ^•^OH reacts with unsaturated bonds such as C=C, C=O, and C=S [32]. The PL response of TPE-C(4)N^+^Me_3_Br^−^ to Fenton’s reagent was solvent-dependent. Figure 5c shows the changes in the PL spectra of aqueous solutions of TPE-C(4)N^+^Me_3_Br^−^ with the addition of Fenton’s reagent. The computational calculations revealed that the PL peaks of TPE at 459 nm and 540 nm corresponded to the S_3_ → S_0_ and S_1_ → S_0_ transitions, respectively [33]. As shown in Figure 5c, the peak at approximately 470 nm, which corresponds to S_1_ → S_0_ transition, disappeared with the addition of four equimolar amounts of Fenton’s reagent. This result is in agreement with the result that the absorption corresponding to the π–π* transitions of TPE-C(4)N^+^Me_3_Br^−^ disappeared after the addition of four equimolar amounts of Fenton’s reagent. The fact that the addition of further amounts of Fenton’s reagent caused the PL peak at 410 nm, which corresponds to the S_3_ → S_0_ transition, to intensify was possibly attributable to the occurrence of AIE. In contrast, the PL intensity of DMSO solutions of TPE-C(4)N^+^Me_3_Br^-^ decreased with increasing amounts of Fenton’s reagent (Figure 5d). Considering reports that ^•^OH reacts with DMSO to yield ^•^CH_3_, the reason for the decrease in the PL in the DMSO solution of TPE-C(4)N^+^Me_3_Br^-^ upon the addition of Fenton’s reagent is unclear. However, the geometry of the excited state of the reaction product of TPE-C(4)N^+^Me_3_Br^−^ and ^•^OH could differ from that of the product of TPE-C(4)N^+^Me_3_Br^−^ and ^•^CH_3_. The extent to which the PL of TPE-C(4)N^+^Me_3_Br^−^ was quenched using Fenton’s reagent was quantified using the Stern–Volmer equation, *I*_0_/*I* = 1 + *K*_SV_[quencher], where *I*_0_ and *I* denote the PL intensities with and without the quencher, respectively. The Stern–Volmer plots for the PL quenching of TPE-C(4)N^+^Me_3_Br^−^, shown in Figure 5e, are convex downward. This result indicates that the PL quenching is attributed to the presence of the combined effect of static and dynamic mechanisms. The occurrence of both static and dynamic quenching for the same fluorophore is represented by a nonlinear equation in terms of the quencher concentration ([Q]): *F*_0_/*F* = (1 + *K*_D_[*Q*])(1 + *K*_S_[*Q*]), where *K*_D_ and *K*_S_ are static and dynamic Stern–Volmer constants, respectively [34]. The modified Stern–Volmer equation is a quadratic function of [*Q*], which accounts for the downward curvature that was observed.

Figure 5f shows the changes in the UV-vis spectra of TPE-C(4)N^+^Me_3_TCNQ^−•^ in DMSO with the addition of Fenton’s reagent. The absorption corresponding to the D_0_ → D_2_ and D_0_ → D_1_ transitions of TCNQ^−•^ of TPE-C(4)N^+^Me_3_TCNQ^−•^ decreased with increasing amounts of Fenton’s reagent. These results indicate that TPE-C(4)N^+^Me_3_TCNQ^−•^ could be used for the detection of ^•^CH_3_ by monitoring the absorption changes. The disappearance of the peak corresponding to the TCNQ^−•^ group indicates that TCNQ^−•^ had reacted with ^•^CH_3_. This assumption is confirmed by the appearance of the peak corresponding to the TCNQ^−^ group at 329 nm. The peak appearance was accompanied by an increase in the absorption at 488 nm probably because of the formation of TCNQ^2−^. TCNQ^2−^, which is known to form via the disproportionation reaction of TCNQ^−^, exhibited an absorption maximum at approximately 500 nm [35]. The UV-vis response of TPE-C(*m*)-N^+^Me_3_TCNQ^−•^ (*m* = 2 and 6) to Fenton’s reagent is expected to be similar to that of TPE-C(4)-N^+^Me_3_TCNQ^−•^ as the UV-vis spectra of TPE-C(*m*)-N^+^Me_3_TCNQ^−•^ (*m* = 2, 4, and 6) are similar irrespective of the chain length, as shown in Figure S3b. The PL response of TPE-C(4)N^+^Me_3_TCNQ^−•^ to Fenton’s reagent in DMSO was considerably different from that of TPE-C(4)N^+^Me_3_Br^−^. The decrease in the PL intensity of TPE-C(4)N^+^Me_3_TCNQ^−•^ with the addition of Fenton’s reagent was considerably less pronounced than that of TPE-C(4)N^+^Me_3_Br^−^ (Figure S6). As mentioned above, the TCNQ^−•^ group of TPE-C(4)N^+^Me_3_TCNQ^−•^ reacted with ^•^CH_3_. However, the bulky TCNQ group may hinder the reaction of the TPE core with ^•^CH_3_, as is evident from the negligible changes in the PL spectra recorded after the addition of Fenton’s reagent.

## 3. Experimental Section

### 3.1. General

TPE-C(*m*)Br, TPE-C(*m*)N^+^Me_3_Br^−^ (*m* = 2, 4, and 6) [36], and Li^+^TCNQ^−•^ [26] were prepared according to the literature. (TTAGGG)*_n_* (*n* = 2, 4, and 6; purified via HPLC) was purchased from Nippon Gene Co. LTD. (Tokyo, Japan). Other reagents were purchased and used without further purification. Acetonitrile, dimethyl sulfoxide (DMSO), methanol, and tetrahydrofuran (THF) were obtained from WAKO Chemical Co. Limited. (Odawara, Japan). These solvents were dried, distilled, and stored under N_2_. Bovine serum albumin (BSA), hydrogen peroxide, and iron(II) sulfate heptahydrate were purchased from WAKO Chemical Co. Limited. (Odawara, Japan). Silver nitrate and 7,7′,8,8′-tetracyanoquinodimethane (TCNQ) were purchased from TCI Co. Limited. (Taipei City, Taiwan). The reactions were carried out using standard Schlenk techniques under nitrogen.

IR and NMR measurements were caried out with a JASCO FT/IR-660 PLUS spectrophotometer with a KBr pellet and JEOL AL-400 spectrometers, respectively. Elemental analysis was conducted on a Yanagimoto MT-5 CHN corder. UV-vis and PL spectra were recorded on a JASCO V-560 spectrometer and a JASCO FP-6200, respectively. Photoluminescence lifetime measurements were conducted with a HORIBA FluoroCube Model:1000U. The CLSM images were obtained using a Leica TCS SP5. Cyclic voltammetry was performed with a Hokuto Denko HSV-110, and 1 cm × 1 cm and 1 cm × 2 cm Pt plates and Pt wire were used as working, counter, and reference electrodes, respectively. Tetraethylammonium tetrafluoroborate was used as an electrolyte. The scan speed was 50 mV·s^−1^.

### 3.2. Synthesis of TPE-C(m)N^+^Me_3_TCNQ^−•^

TPE-C(4)Br (0.15 g, 0.12 mmol) was dissolved in 15 mL of dry MeOH at 80 °C. After Li^+^TCNQ^−•^ (0.16 g, 0.75 mmol) was added to the solution, the reaction solution was stirred at 25 °C for 36 h. The precipitate was collected via filtration and dried under vacuum to afford TPE-C(4)N^+^Me_3_TCNQ^−•^ as a blue purple powder (0.16 g, 80%). ^1^H NMR (400 MHz, DMSO-*d*_6_): δ 6.86 (8H), 6.71 (8H), 3.99 (8H), 3.34 (8H), 3.05 (36H), 1.82 (8H), 1.71 (8H). Calcd. for (C_102_H_100_N_20_O_4_•0.6H_2_O): C, 72.89; H, 6.07; N, 16.67. Found: C, 72.85; H, 5.79; N, 16.49.

TPE-C(2)N^+^Me_3_TCNQ^−•^ and TPE-C(6)N^+^Me_3_TCNQ^-.^ were synthesized in a similar manner. Data of TPE-C(2)N^+^Me_3_TCNQ^−•^: blue purple powder. Yield = 60%. ^1^H NMR (400 MHz, DMSO-*d*_6_): δ 6.90 (8H), 6.78 (8H), 4.35 (8H), 3.73 (8H), 3.15 (36H). Calcd. for (C_94_H_92_N_20_O_4_•0.3H_2_O): C, 71.85; H, 5.94; N, 17.83. Found: C, 71.83; H, 5.09; N, 17.36. Data of TPE-C(6)N^+^Me_3_TCNQ^−•^: Blue purple powder. Yield = 42%. ^1^H NMR (400 MHz, DMSO-*d*_6_): δ 6.82 (8H), 6.67 (8H), 3.87 (8H), 3.26 (8H), 3.03 (36H), 1.69 (16H), 1.44 (8H), 1.33 (8H). Calcd. for (C_110_H_116_N_20_O_4_•H_2_O): C, 73.39; H, 6.61; N, 15.56. Found: C, 73.41; H, 5.96; N, 14.84.

## 4. Conclusions

In conclusion, the chemical properties and applicability as biomolecule sensors of the TPE derivatives bearing alkylammonium bromide (TPE-C(*m*)N^+^Me_3_Br^−^) and the alkylammonium TCNQ radical anion (TPE-C(*m*)N^+^Me_3_TCNQ^−•^) were investigated. PL measurements revealed that TPE-C(*m*)N^+^Me_3_Br^−^ aggregated in DMSO/THF mixtures, and their PL intensities increased as the THF content increased. The CV measurements indicated that the TPE derivatives obtained in this study were electrochemically active. TPE-C(*m*)N^+^Me_3_Br^−^ could be used as sensors for BSA and telomere DNA by detecting the changes in their PL intensities upon the addition of the analytes. The sensing performance of TPE-C(*m*)N^+^Me_3_Br^−^ depended on the length of the alkyl chain of TPE-C(*m*)N^+^Me_3_Br^−^. Anionic contaminants in sample solutions may affect the sensing performance of TPE-C(*m*)-N^+^Me_3_Br^−^ because the sensors are based on the occurrence of AIE, attributed to the interaction between the cationic group of TPE-C(*m*)N^+^Me_3_Br^−^ and the anionic group of BSA and DNA. In the future, we will study the analyte selectivity of the sensors via an investigation of the optical responses of TPE-C(*m*)-N^+^Me_3_Br^−^ to the addition of BSA or DNA in the presence of various anionic species. TPE-C(*m*)N^+^Me_3_Br^-^ and TPE-C(4)N^+^Me_3_TCNQ^−•^ were also found to be suitable for use as sensors for radical species, as the changes in the intensity of the UV-vis and PL peaks were dependent on the amount of Fenton’s reagent. We can conclude that the results of this study indicated that TPE derivatives bearing alkylammonium salts can be used as sensing materials for biological and radical species.

## Data Availability

Not applicable.

## Supplementary Materials

The following supporting information can be downloaded at: https://www.mdpi.com/article/10.3390/molecules28155663/s1. Figure S1: IR spectra of TPE-C(*m*)N^+^Me_3_TCNQ^−•^ (*m* = 2, 4, and 6). Figure S2: ^1^H NMR spectra of TPE-C(*m*)N^+^Me_3_TCNQ^−•^ (*m* = 2, 4, and 6) in DMSO-*d*_6_. Figure S3: UV-vis spectra of TPE-C(*m*)N^+^Me_3_Br^−^ and TPE-C(*m*)N^+^Me_3_TCNQ^−•^ (*m* = 2, 4, and 6) in DMSO. Figure S4: Plots for the peak currents of the oxidation of Br^−^ to Br_3_^−^ (*i*_pa_(2)) and reduction of Br_3_^−^ to Br^−^ (*i*_pc_(2)) vs. *v*^1/2^. Figure S5: CV curves of (a) TPE-C(2)N^+^Me_3_TCNQ^−•^ and (a) TPE-C(6)N^+^Me_3_TCNQ^−•^ in acetonitrile containing 0.10 M [Et_4_N]BF_4_. Figure S6: PL spectral changes observed for TPE-C(*m*)N^+^Me_3_Br^−^ (*m* = 2 and 6) upon the addition of BSA in 0.1 equimolar amounts. Figure S7: Images of interactions between (a) BSA and TPE-C(2)N^+^Me_3_Br^−^, (b) BSA and TPE-C(6)N^+^Me_3_Br^−^, (c) (TTAGGG)*_n_* and TPE-C(2)N^+^Me_3_Br^−^, and (d) (TTAGGG)*_n_* and TPE-C(6)N^+^Me_3_Br^−^. Figure S8: PL spectral changes observed for TPE-C(*m*)N^+^Me_3_Br^−^ (*m* = 2 and 4) upon the addition of (TTAGGG)_6_ in 0.1 equimolar amounts. Figure S9: PL spectral changes observed for the DMSO solutions of TPE-C(4)N^+^Me_3_TCNQ^−•^ upon the addition of Fenton’s reagent.

## Sample Availability

Samples of the compounds are not available from the authors.
