# Supplementary material for: Tetraphenylethene Derivatives Bearing Alkylammonium Substituents: Synthesis, Chemical Properties, and Application as BSA, Telomere DNA, and Hydroxyl Radical Sensors
Source: Molecules. 2023 Jul 26;28(15):5663. doi: 10.3390/molecules28155663 (PMC10419492; doi:10.3390/molecules28155663)
Supplement: Supplementary file 1 [file molecules-28-05663-s001.zip › molecules-2525976-supplementary.pdf]

Supporting Information

Contents

Figure S1. IR spectra of TPE-C(m)N$^+$Me$_3$TCNQ$^{-\bullet}$ (m = 2, 4, and 6). 2

Figure S2. ^{1}H NMR spectra of TPE-C(m)N$^+$Me$_3$TCNQ$^{-\bullet}$ (m = 2, 4, and 6) in DMSO-d_6. 3

Figure S3. UV-vis spectra of TPE-C(m)N$^+$Me$_3$Br$^-$ and TPE-C(m)N$^+$Me$_3$TCNQ$^{-\bullet}$ (m = 2, 4, and 6) in DMSO. 4

Figure S4. Plots for the peak currents of the oxidation of Br$^-$ to Br$_3^-$ (i_{pa}(2)) and reduction of Br$_3^-$ to Br$^-$ (i_{pc}(2)) vs. $v^{1/2}$. 5

Figure S5. CV curves of (a) TPE-C(2)N$^+$Me$_3$TCNQ$^{-\bullet}$ and (a) TPE-C(6)N$^+$Me$_3$TCNQ$^{-\bullet}$ in acetonitrile containing 0.10 M [Et$_4$N]BF$_4$. 5

Figure S6. PL spectral changes observed for TPE-C(m)N$^+$Me$_3$Br$^-$ (m = 2 and 6) upon the addition of BSA in 0.1 equimolar amounts. 6

Figure S7. Images of interactions between (a) BSA and TPE-C(2)N$^+$Me$_3$Br$^-$, (b) BSA and TPE-C(6)N$^+$Me$_3$Br$^-$, (c) (TTAGGG)$_n$ and TPE-C(2)N$^+$Me$_3$Br$^-$, and (d) (TTAGGG)$_n$ and TPE-C(6)N$^+$Me$_3$Br$^-$. 7

Figure S8. PL spectral changes observed for TPE-C(m)N$^+$Me$_3$Br$^-$ (m = 2 and 4) upon the addition of (TTAGGG)$_6$ in 0.1 equimolar amounts. 8

Figure S9. PL spectral changes observed for the DMSO solutions of TPE-C(4)N$^+$Me$_3$TCNQ$^{-\bullet}$ upon the addition of Fenton's reagent. 8

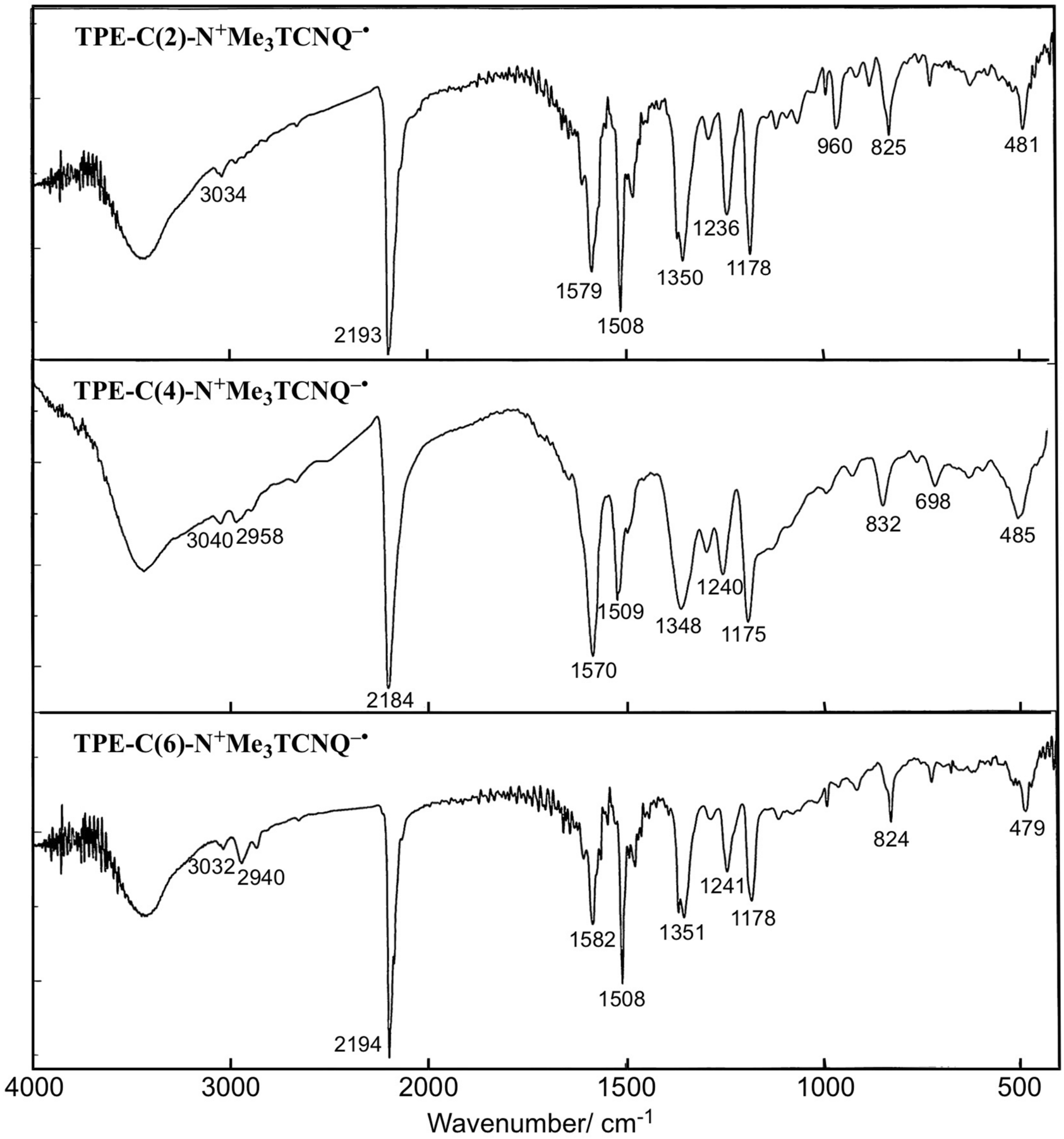

Figure S1. IR spectra of TPE-C(m)$N^+Me_3TCNQ^{-\bullet}$ (m = 2, 4, and 6).

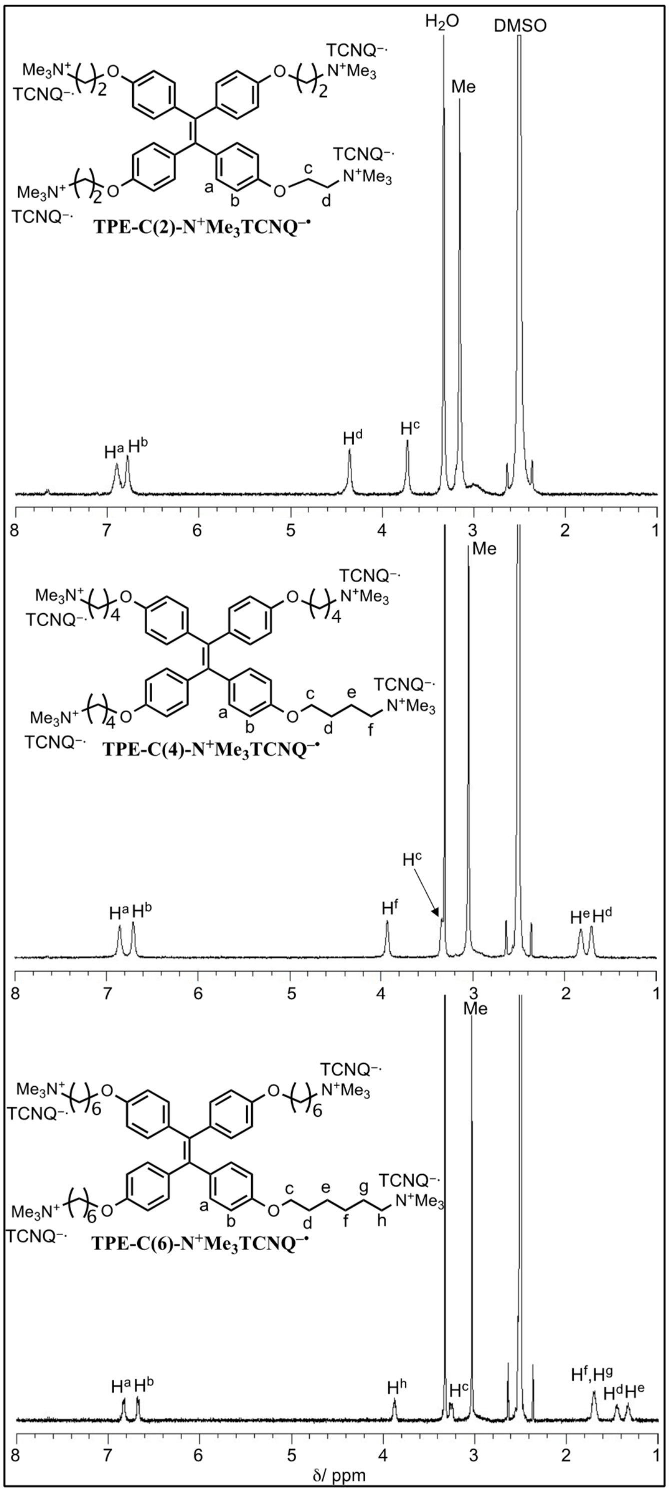

Figure S2. ^{1}H NMR spectra of TPE-C(*m*)N^{+}Me$_3$TCNQ$^{-\bullet}$ (*m* = 2, 4, and 6) in DMSO-*d*$_6$.

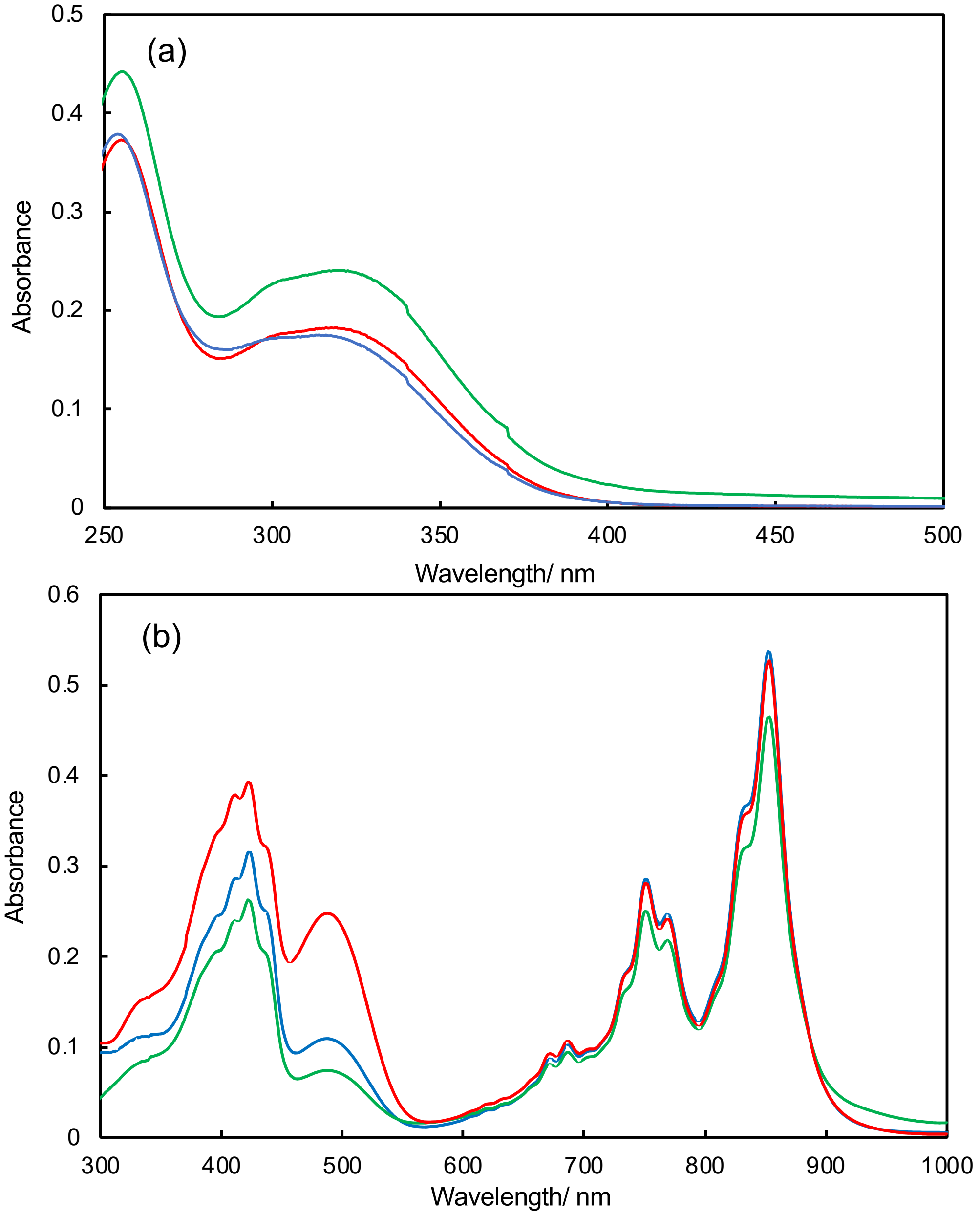

Figure S3. UV-vis spectra of TPE-C(*m*)$N^+Me_3Br^-$ and TPE-C(*m*)$N^+Me_3TCNQ^{-\bullet}$ (*m* = 2: blue curve, 4: red curve, and 6: green curve) in DMSO.

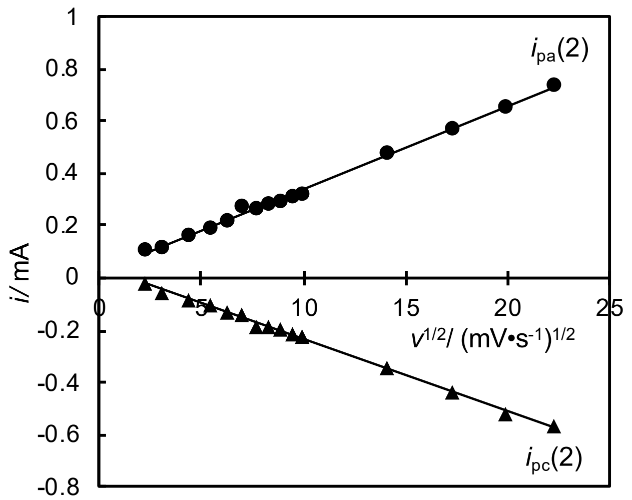

Figure S4. Plots for the peak currents of the oxidation of Br^- to Br_3^- ($i_{pa}(2)$) and reduction of Br_3^- to Br^- ($i_{pc}(2)$) vs. $v^{1/2}$.

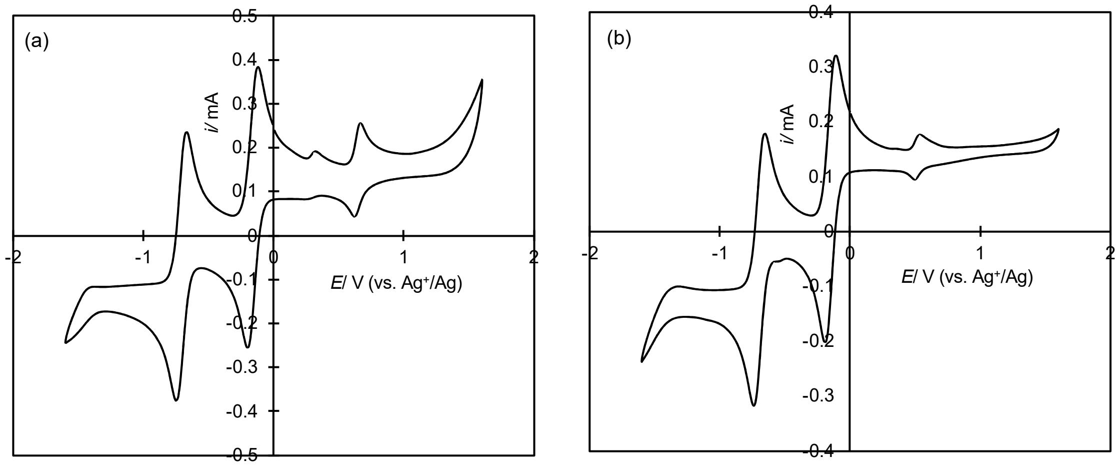

Figure S5. CV curves of (a) TPE-C(2)$N^+Me_3TCNQ^{-\bullet}$ and (b) TPE-C(6)$N^+Me_3TCNQ^{-\bullet}$ in acetonitrile containing 0.10 M $[Et_4N]BF_4$.

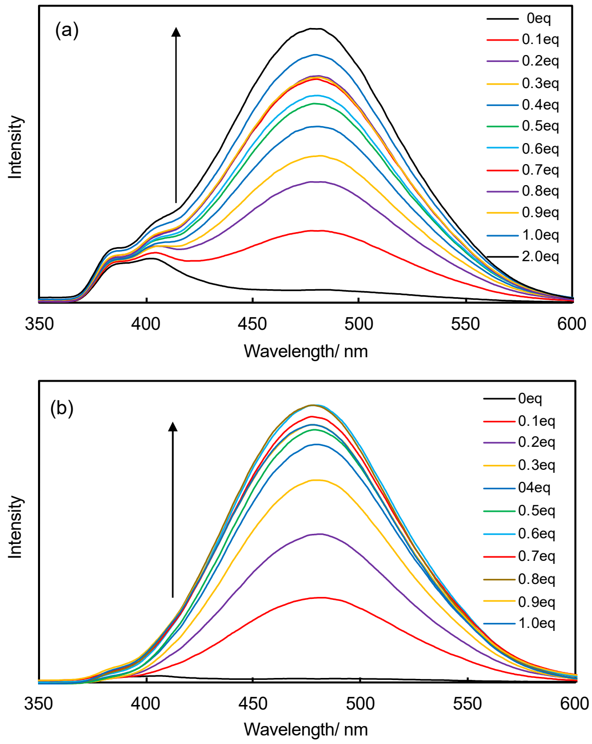

Figure S6. PL spectral changes observed for (a) TPE-C(2)$N^+Me_3Br^-$ and (b) TPE-C(6)$N^+Me_3Br^-$ upon the addition of BSA in 0.1 equimolar amounts.

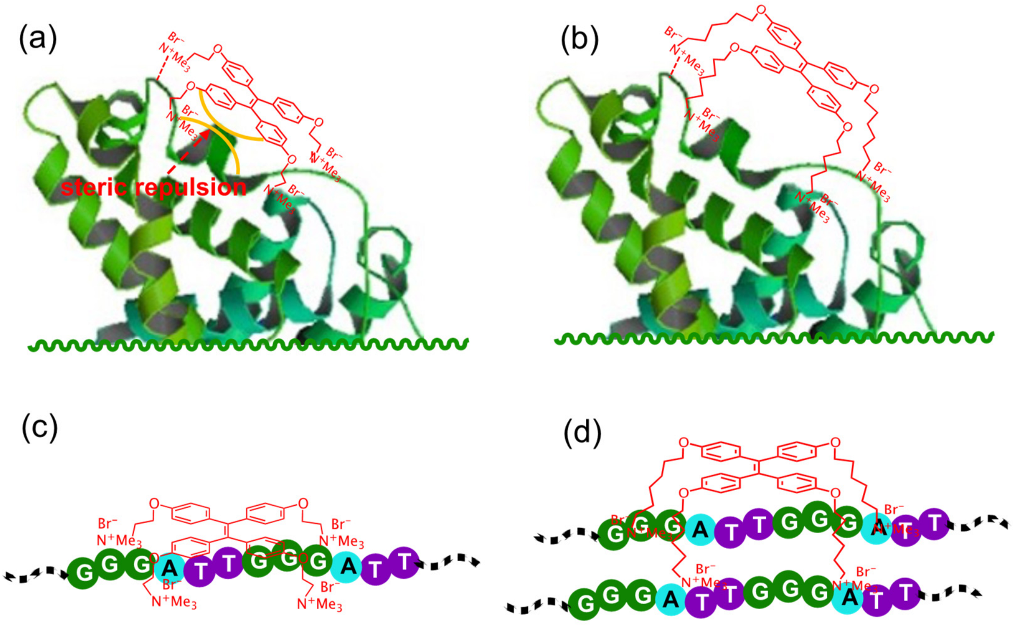

Figure S7. Images of interactions between (a) BSA and TPE-C(2)$N^+Me_3Br^-$, (b) BSA and TPE-C(6)$N^+Me_3Br^-$, (c) $(GGGATT)_n$ and TPE-C(2)$N^+Me_3Br^-$, and (d) $(GGGATT)_n$ and TPE-C(6)$N^+Me_3Br^-$. These interactions induce the inhibition of bond rotation between the phenyl and ethene groups, which causes AIE

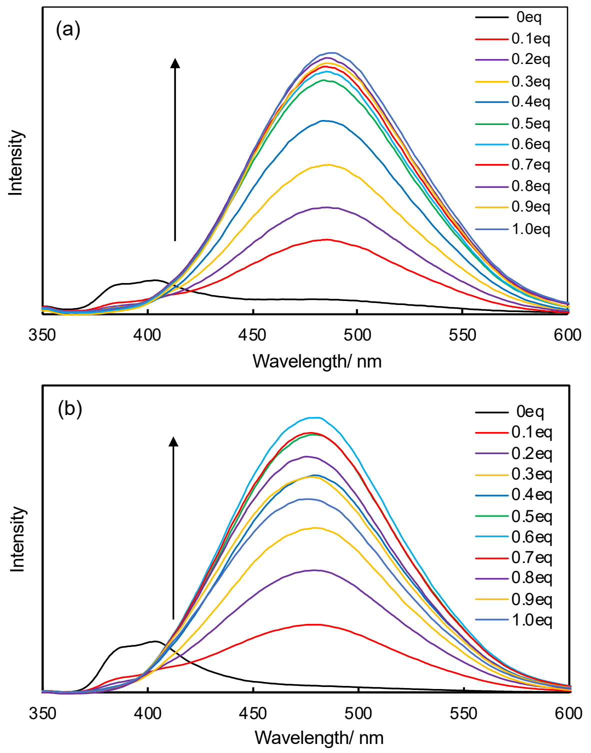

Figure S8. PL spectral changes observed for (a) TPE-C(2)N$^+$Me$_3$Br$^-$ and (b) TPE-C(4)N$^+$Me$_3$Br$^-$ upon the addition of $(TTAGGG)_6$ in 0.1 equimolar amounts.

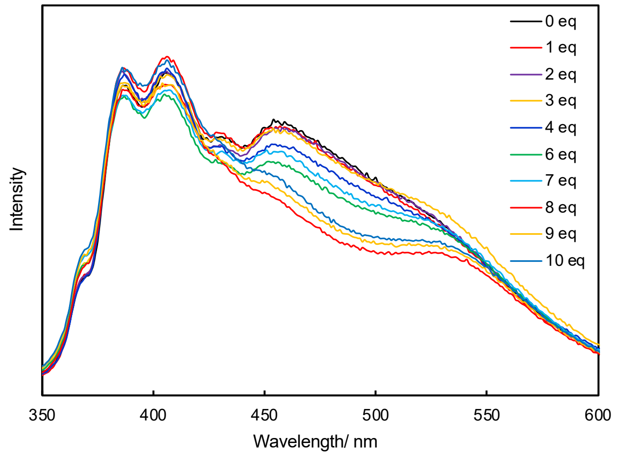

Figure S9. PL spectral changes observed for the DMSO solutions of TPE-C(4)N$^+$Me$_3$TCNQ$^{-\bullet}$ upon the addition of Fenton's reagent.
